# Content and Factors Influencing Health Education in Immunisation Clinics in Nigeria

**DOI:** 10.1111/tmi.70101

**Published:** 2026-02-08

**Authors:** Osayame A. Ekhaguere, Nnette Ekpenyong, Esosa F. Mohammed, Fatima Lawan Bukar, Angela Oyo‐Ita, Jessica Kaufman

**Affiliations:** ^1^ Department of Pediatrics Indiana University School of Medicine Indianapolis Indiana USA; ^2^ Department of Community Health University of Calabar Calabar Cross River State Nigeria; ^3^ Ballad Health Johnson City Tennessee USA; ^4^ Department of Community Medicine University of Maiduguri Teaching Hospital Maiduguri Borno State Nigeria; ^5^ Vaccine Uptake Group, Murdoch Children's Research Institute Parkville Australia; ^6^ Faculty of Medicine, Dentistry and Health Sciences The University of Melbourne Parkville Australia

**Keywords:** immunisation communication, low‐ and middle‐income countries, vaccine communication, vaccine communication content, vaccine communication in group settings

## Abstract

**Background:**

Most parents in Nigeria receive childhood vaccine education in a group clinic setting. These health talks are not standardised, and little is known about the topics addressed, how these topics are chosen, and how parents experience these talks. This study aimed to fill this knowledge gap.

**Method:**

We conducted a qualitative study in eight immunisation clinics across rural and urban sites in Borno and Cross River State, Nigeria. We audio‐recorded two health education talks at each clinic—one with the health worker unaware of being recorded. We interviewed 40 parents and 16 health workers to gather insights. Content analysis identified topics covered, while thematic analysis explored motivations for the health education content from health workers' and parents' perspectives on the talks.

**Results:**

The content and delivery method of the recorded health talks varied considerably within and between different health workers. Of the 16 analysed talks, 6 (37.5%) contained non‐vaccine‐related topics such as breastfeeding and family planning. The content of health talks was based on a clinic topic schedule, prevailing interests such as ongoing disease outbreaks, or the health workers' feelings and moods. Health workers mentioned they receive little to no formal training on the content and delivery of vaccine education. Current competency was primarily based on training received during formal schooling, embedded within other health programs or media outlets aimed at the general population. Parents generally perceived the educational sessions to be informative and important. However, short health talks, long clinic sessions and a lack of courtesy were barriers to a positive clinic experience.

**Conclusion:**

Inadequate and inconsistent health education on immunisation is prevalent at group‐setting vaccine clinics in Nigeria and is driven by a lack of health worker training. Research and policy changes are critically needed to improve the quality of vaccine health education.

## 1 Background

Routine childhood immunisations are a cost‐effective public health intervention [1]. However, despite concerted local and global efforts, childhood immunisation coverage has stagnated, with approximately 21 million children remaining unvaccinated or under‐vaccinated globally, largely in low‐ and middle‐income countries (LMICs) in sub‐Saharan Africa and South Asia [2, 3, 4]. Nigeria is among the 10 countries with the lowest childhood vaccine uptake, contributing 15% of the world's zero‐dose children [5], and had the third diphtheria‐tetanus‐pertussis vaccine (DPT3) completion rate of 60% in 2023 [3]. Furthermore, substantial within‐country regional variation exists, with the southern region having a two‐to‐three‐fold higher immunisation completion rate than the northern region [6].

Barriers to childhood vaccine uptake arise from complex behavioural and social factors, which WHO categorises into four domains: thinking and feeling, motivation, social processes and practical issues [7]. These domains encompass parents' perceptions of vaccine‐preventable diseases, beliefs about safety, efficacy and trust in vaccines, social influences such as family norms and health worker recommendations, and logistical challenges like accessibility and service quality [7]. Successful childhood immunisation programs rely on parents and caregivers (henceforth referred to as parents) to have timely, accurate, culturally appropriate information to build sufficient knowledge, awareness and acceptance of immunisation to decide to vaccinate their child [8, 9]. Effective communication enhances parental self‐efficacy and reduces vaccine hesitancy to improve routine childhood immunisation [10, 11, 12, 13].

In Nigeria, communications about immunisations occur primarily at immunisation clinics [14, 15, 16]. Compared to the one‐on‐one provider‐patient interaction common in high‐income countries, immunisation education in Nigeria typically occurs in group settings, where a health worker delivers an immunisation health talk to an assembly of parents during clinic sessions. A review of the literature indicates that the duration of this method of health talk can vary widely, from 2 min to over 1 h [16, 17, 18], suggesting that considerable differences may exist in what is communicated to parents. However, there is limited evidence on the exact content of the health talks in group clinic settings in Nigeria to inform interventions to improve effective immunisation communication. Thus, this study aimed to (a) describe the topics discussed in immunisation health talks, (b) explore variation in topics within the same and between different health workers, (c) understand the factors that determine which topics are discussed and (d) examine parental perspectives on the immunisation health talk received during group settings in immunisation clinics in Nigeria.

## 2 Method

### 2.1 Study Design and Setting

**FIGURE 1:**
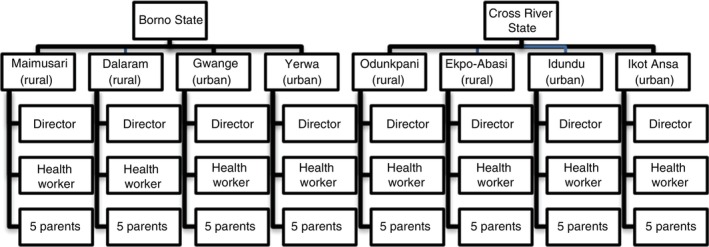
Distribution of study participants by state, study site and category.

We conducted a qualitative descriptive study to explore the contextual nuances of health talks and the experiences of those delivering and receiving these talks. We interviewed parents and healthcare workers individually and recorded immunisation health education talk sessions. The study was conducted in two Nigerian states: Borno in the northeastern region and Cross River in the southeastern region. In each state, we selected four immunisation clinics (two urban and two rural), totalling eight study sites (Figure 1). Site selection was conducted in consultation with the State Health Ministry and the Primary Health Care Board, which oversees immunisation activities at the local level to ensure representation from historically high‐ and low‐performing immunisation clinics.

The National Primary Health Care Development Agency oversees vaccine provision and immunisation guidelines, while the National Social Mobilisation Working Group develops communication strategies for vaccination programs. State and Local Committees implement these interventions [19]. Vaccines are primarily administered at immunisation clinics, with supplementary community immunisation campaigns. The national immunisation schedule recommends completing all routine childhood vaccinations by 15 months [20].

In Nigeria, the DPT3 coverage rate is 62%, with significant regional disparities [21]. Borno State, predominantly Islamic, has a coverage rate of 30%, while Cross River State, predominantly Christian, has 75% [6].

### 2.2 Study Participants and Recruitment

The study targeted two participant groups (Figure 1). The first group consisted of health workers responsible for delivering health education and coordinating clinic activities. These include certified nurses, public health workers, clinic directors and community health extension workers. The second study group was parents visiting the clinic for child immunisation services who listened to the immunisation health talk.

Following ethical approval, we sought permission from each clinic's director to recruit health workers and parents. We purposively recruited all health workers at the clinic on the day the study was conducted and used convenience sampling to enrol parents. For both groups, the study team explained the study purpose and procedure and obtained signed informed consent at the beginning of the clinic session. No participant dropped out, and we did not collect demographic information on parents who refused to participate.

### 2.3 Study Procedures

*Recording vaccine health education talk*: We audio‐recorded a health worker delivering a vaccine health education talk twice, at least 1 week apart. One recording used the secret shopper technique, where the worker was unaware that they were being recorded. This method involves researchers posing as patients to gain unbiased insights into healthcare delivery, revealing barriers that traditional surveys might miss [22]. The health worker was informed after the recording so they could consent to its use.

*Individual interviews*: We conducted semi‐structured interviews with the health workers, clinic directors and parents who received the recorded immunisation talk. For health workers, we sought to understand the training and motivation for the health talk content. For the parents, we sought to understand their perception of the vaccine's health content and delivery. Two research assistants conducted the interviews using a pre‐developed, pre‐tested interview guide. The interviews were conducted in English, Pidgin English, and the local dialect, lasting 30–45 min. Study participants were only interviewed once. The interviews were conducted by a trained research assistant or senior public health trainee trained in qualitative interviewing. The interviews were audio recorded, and notes were taken. Interviews conducted in local languages were translated and transcribed into English by the same trained research assistants who conducted the interview, who were fluent in both the source language and English. To preserve participant meaning, transcripts were reviewed alongside audio recordings, and ambiguous phrases were discussed among the research team to ensure contextual accuracy.

Thematic saturation was assessed iteratively during data analysis. Saturation was considered achieved when no new codes or themes emerged in successive interviews. Specifically, after coding approximately the first 30 parent interviews and 12 health worker interviews, no additional substantive codes were identified in the remaining transcripts, indicating adequate thematic coverage; therefore, no further interviews were conducted. The interviews were conducted in a quiet office provided by the clinic directors. Parents and health workers received $10 and $20 for their time and effort, respectively. The study activities occurred between October and November 2023.

### 2.4 Data Analysis

We used content analysis to analyse each health talk (21). Due to limited immunisation health education content recommendations in a group setting, we first developed a checklist with all immunisation‐specific topics mentioned in any health talk (Supplemental Table S1). The checklist categories included vaccine name, age of vaccine administration, diseases vaccines prevent, administration site of vaccines, typical side effects, how to manage side effects, vaccination with mild illness, and the importance of the vaccination card. Second, we appraised individual talks based on this list.

Given that specific immunisations, such as the Pentavalent vaccine, are administered at different time points (6, 10 and 14 weeks) if the health worker referred to a previously mentioned vaccine, we included the topic they referenced. For example, if the health worker talked about Penta 1, which is given at 6 weeks, and while talking about the 10‐week scheduled vaccine, she made a statement such as ‘At the ten weeks, you will receive the same vaccine you received at six weeks’, we reported that Penta 2 was discussed.

Non‐vaccine‐related topics were also documented. To determine variations in content, we tabulated key content within and between health workers. We categorised the delivery style into interactive if the health worker engaged in back‐and‐forth conversations with parents while delivering the health talk and non‐interactive otherwise. For talks that had non‐vaccine‐related topics, we compared the word count of vaccine‐related and non‐vaccine‐related topics.

We used a descriptive content analysis approach to analyse the interviews [23]. Starting with an initial set of codes based on research questions, we categorised health workers' inputs on policies, procedures, training and values influencing health talks, while focusing on parents' perceptions of their value. To enhance analytic rigour, OAE and EFM independently coded initial transcripts using a shared coding framework and met regularly to discuss discrepancies and refine code definitions through consensus. This coding framework was refined through iterative readings, resulting in a tree with two main branches: (1) Factors informing health talk delivery and (2) Parent experiences, which included both deductive and inductive themes. O.A.E. and E.F.M. applied this framework to all transcripts, while senior authors A.O.‐I. and J.K. reviewed major codes when necessary. Formal inter‐rater reliability statistics were not calculated, consistent with qualitative analytic approaches that prioritise depth, reflexivity and consensus over numerical agreement metrics. All coding was performed manually using Excel, and transcripts were not returned to participants for review.

To maintain site anonymity, results are reported using pseudonyms that represent states, and if the site was rural or urban.

### 2.5 Ethical Approval

The Borno, Cross Rivers State and Indiana University institutional review board ethical review board approved the study (Protocol # 97/2023, # CRSMOH/HRP/REC/2023/416 and # 20006). Each study participant gave written informed consent before any study activity.

## 3 Results

### 3.1 Content Analysis of Health Talks

**FIGURE 2:**
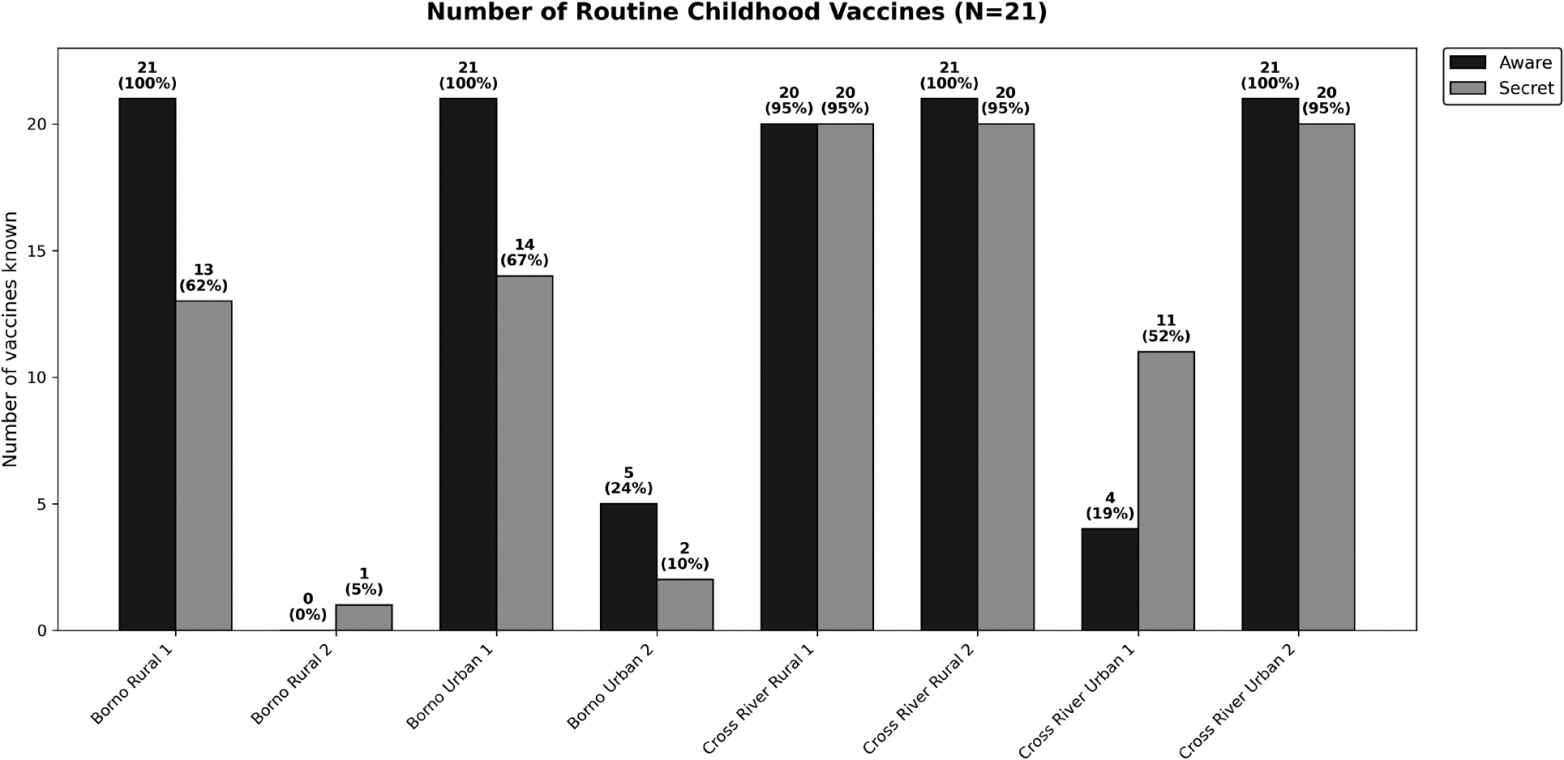
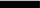
Assessment, while providers were aware they were being recorded. 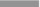
Assessment, while providers were unaware, they were being recorded (secret shopper).

We analysed 16 immunisation health talks from eight clinics, averaging 11 min in length (SD 5 min). Most talks (61%) mentioned 10 or more of the 21 vaccines in the childhood immunisation schedule, with sites often naming more vaccines when aware they were being recorded. Only one talk (12%) from Cross River State mentioned 10 or fewer vaccines, compared to four (50%) in Borno State (Figure 2).

**FIGURE 3:**
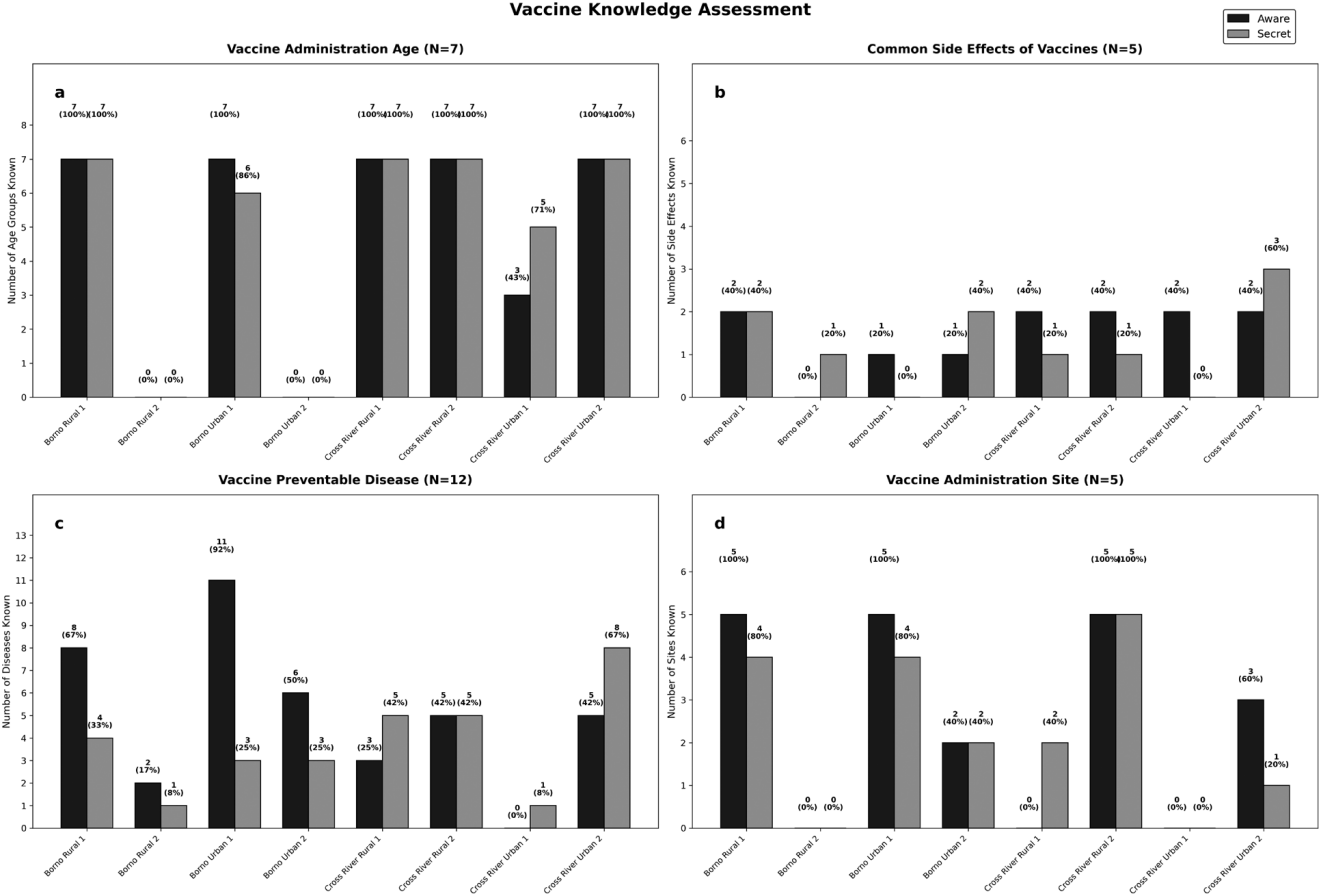
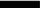
Assessment, while providers were aware they were being recorded. 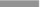
Assessment, while providers were unaware, they were being recorded (secret shopper).

The age of immunisation was the topic with the least variation. However, 25% of talks did not mention this topic (Figure 3a). Half of the talks noted two or more common vaccine side effects, while 12.5% did not mention any. Four talks (25%) mentioned six or more vaccine‐preventable diseases (Figure 3c), and seven covered three or more vaccine administration sites (Figure 3d).

Additionally, 12.5% mentioned that vaccines can be safely administered during mild illnesses, 37.5% addressed side effect management and 50% highlighted the importance of vaccine cards. An interactive communication style was used in only 12.5% of talks, primarily when workers knew they were being recorded. Ten talks (62.5%) focused solely on vaccines; others included topics like family planning, breastfeeding, general hygiene and nutrition.

### 3.2 Thematic Analyses of Health Workers and Patient Interviews

We interviewed one director, a healthcare worker, and five parents at each site. Their demographic characteristics are provided in Table 1.

**TABLE 1:** Study participants' demographics.

Directors	
Characteristic	*N* = 8
Age, years, mean (SD)	44 (10)
Education, *n* (%)*	
University Diploma	1 (12.5)
Bachelor's degree or higher	5 (62.5)
Missing	2 (25)
Years of practice, mean (SD)	19 (11)

Healthcare workers	
Characteristic	*N* = 8
Age, years, mean (SD)	43 (10)
Education, *n* (%)	
University Diploma^a^	1 (12.5)
Bachelor's degree or higher	7 (87.5)
Years of practice, mean (SD)	18 (10)

Parents*	
Characteristic	*N* = 40
Age, years, mean (SD)	28 (6)
Average number of children, (SD)	3 (2)
Education, *n* (%)	
No or primary education	8 (20)
Secondary Education	23 (57.5)
Tertiary education	8 (20)
Missing	1 (2.5)
Occupation	
Homemaker	8 (20)
Self‐employed or petty trader	24 (60)
Formally employed	6 (15)
Student	2 (5)

* All interviewed parents and health workers were female.

^a^ Post‐secondary diploma includes non‐degree health training programs awarded by universities (e.g., diplomas or postgraduate diplomas) or by health training institutions (e.g., Community Health Extension Worker diplomas), which are distinct from bachelor's or postgraduate university degrees.

The major themes and supporting quotes from health workers.

#### 3.2.1 Factors Informing Health Talk Delivery

The interviews with clinic directors revealed that they or the health workers did not receive formal training on vaccine communication. Instead, they gained knowledge through broader primary health care training programs, such as the Basic Emergency Obstetric and Newborn Care and the Integrated Management of Childhood Illness. Additional training opportunities came from non‐governmental organizations (NGOs) and during routine local Ministries of Health supervisory visits. They also received no structured education on communication techniques for engaging with parents in a group setting.

As one director explained, training was delivered through multiple channels, depending on circumstances:

These trainings were given to us in many ways, that is, those that had it during their school days or our in‐house staff that were opportune to receive the training during supportive supervision. (Borno Director, 57 years, 22 years of experience)

Other directors described their sources of vaccine communication education as general monthly meetings and ad‐hoc workshops organised by local government authorities and partner organisations. One director shared:

Every end of the month, we go for an LGA meeting, and they talk about health topics, especially the supporting partners. Even last time, we had a workshop on diphtheria case management at LGA. (Borno Director, 37 years, 15 years of experience)

In the absence of formal training, some directors emphasised a reliance on self‐learning and on‐the‐job experience. As one director succinctly put it:

We train ourselves; it's on‐the‐job training. (Cross River Director, 50 years, 30 years of experience)

Health workers also emphasised the role of experience and peer learning in shaping their communication about vaccines to parents. As one provider described:

I have been practicing these things over the years, so everything is upstairs. (Cross River health worker, 38 years, 8 years of experience).

As your colleague is giving [a talk], the one you don't know, you learn from that colleague. (Cross River health worker, 35 years, 12 years of experience)

#### 3.2.2 Factors Influencing Topic Selection for Health Talks

When asked how they decide on topics for health talks, health workers described a variety of influences, including clinic timetables, seasonal health trends, current epidemics and parental concerns. One provider explained:

I mostly follow the timetable for the weekly activities, but if there is an ongoing epidemic, we can talk about that as well. We also look at the season and diseases that are more common during that time and give health talks on them. (Borno health worker, 41 years, 17 years of experience)

Others tailored their discussions based on the reactions and requests of parents. One provider detailed their approach, saying:

I look at their composure. When I analyze the crowd, I may then ask them what we should discuss. Before I start, I can ask if they have questions. I ask what they think causes disease… So, from this, I can choose from what they have little knowledge of. (Borno health worker, 27 years, 6 years of experience)

#### 3.2.3 Consistency and Variability in Health Talk Delivery

While most health workers stated that they delivered the same content each time, some acknowledged that variations were inevitable. One health worker confidently stated:

I give the exact information every time, yes [she laughs]. We do remember, like the six key messages on immunization – it's very important, it is [posted] on the wall. (Cross River health worker, 38 years).

However, another health worker challenged this idea, asserting:

Honestly, it can never be the same information. (Borno health worker, 27 years, 6 years of experience)

The major themes and supporting quotes from health workers.

All parents had a positive view of the health education sessions, noting similarities to information from antenatal visits, community outreach and immunisation initiatives. Media, particularly TV and radio, also reinforced health messages, though some parents noted inconsistencies, especially regarding specific disease outbreaks. While most were satisfied with the chance to ask questions, some faced barriers such as noise, delays, short talks, long clinic waits and discourteous health workers.

### 3.3 Barriers to a Positive Clinic Experience

Several parents expressed concerns about discourteous health workers. One parent noted that the speaker's delivery affected the experience:

Well, for today's health talker, the lady was not courteous and talked to herself all through. (Borno Mother, 25 years, primary school education, two children)

Timing was another commonly cited barrier, with one parent explaining:

I think it should be earlier. Like today, I came in around 9 o'clock, and the health talk did not start until a few minutes to 11. (Cross River Mother, 29 years, no formal education, three children)

### 3.4 Facilitators of a Positive Clinic Experience

Despite these barriers, several factors contributed to a positive experience. Many parents appreciated that health workers delivered talks in their local dialects, making the information accessible to a broader audience. One mother highlighted this, stating:

The nurses and health workers do the talks in our local dialect, and they are very simple and clear. But if someone does not understand the language, they also repeat it in other languages for them. (Borno Mother, 25 years, primary school education, four children)

Another mother praised the way the health worker simplified the information:

The way she delivered it—she broke it down to layman's understanding. She used both English and vernacular. (Cross River Mother, 22 years, university student, one child)

## 4 Discussion

The content and delivery method of vaccine health education varies considerably within the same health worker and among different health workers in a group clinic setting in Nigeria. Interviews with health workers suggest there is limited structured training on communicating vaccine education in group clinic settings. Instead, their knowledge comes primarily from formal schooling, NGO initiatives and media sources like radio and television. Despite these limitations, parents generally perceive the educational sessions as informative and valuable. However, short health talks, prolonged clinic sessions and a lack of courtesy emerged as barriers to a positive clinic experience.

A review of health worker training manuals produced by WHO (Communicating with Parents and Communities) [24, 25] and UNICEF (interpersonal Communication for Immunisation package) revealed a lack of standardised guidelines on vaccine education content for group clinic settings [26, 27]. The 1997 WHO manual was the only document that provided structured guidance on what the messages should contain, outlining five essential messages that should be communicated to parents: (i) the date and time for the next immunisation appointment, (ii) the place of the next appointment, (iii) the number of remaining visits a child still needs to be fully immunised, (iv) side effects that may occur and (v) how to treat side effects [24].

When taken together (all health talks in this study), the content analysis we performed showed that each health talk mentioned at least one of the following; where on the immunisation card the next vaccine appointment is written, the names and ages (time) for the subsequent immunisation, the most common side effects, and how to treat side effects. However, there was considerable variation in which component of the five essential messages was mentioned, and several health talks failed to mention one or more essential messages. Of the five essential vaccine messages, the place of the next appointment was the only message not mentioned in any health talk. However, it can be assumed that it was understood that the place for the next immunisation appointment would be the same vaccine clinic based on the vaccine delivery organisational structure.

The aforementioned WHO and UNICEF trainings focus on one‐to‐one health worker‐to‐parent interaction, which is not the norm in Nigeria and other LMICs in sub‐Saharan Africa [25, 26, 27]. While the WHO manual does include a section on community‐based communication, these guidelines focus on household visits and community meetings rather than the more common group‐based clinic setting. Importantly, community‐based interventions often target unvaccinated ‘zero‐dose’ children, whose parents may differ in beliefs and demographics from under‐vaccinated parents attending clinic‐based immunisation sessions [28, 29, 30]. Research from LMICs overwhelmingly prioritises community strategies over clinic‐based approaches. A 2023 Cochrane review of interventions to improve childhood immunisations in LMICs revealed that eight of nine studies on parental education took place in community settings [31]. The only trial conducted in the clinic setting provided educational intervention for individuals, not groups [32]. This research gap underscores the need to tailor communication strategies for clinic‐based group education sessions, given that even vaccine‐hesitant parents continue to bring their children for routine immunisation.

The factors influencing the content of health talks varied among study sites. While most health workers tailored their messages based on the mood of the day, ongoing public health concerns (e.g., pandemics) or personal preference, a few followed a set schedule. A key challenge with these ad hoc approaches is the risk of omitting critical information or key information relevant to specific groups of patients. Given that a child will attend seven vaccine clinic visits and must receive 22 different vaccines to protect against 13 vaccine‐preventable diseases, there is a low likelihood that all essential topics will be covered comprehensively over time. This inconsistency reinforces the need for standardised, structured guidelines on vaccine health education content for group clinic settings.

Importantly, not all variability in health talk content should be seen as a flaw. Some differences may be helpful, showing appropriate adjustments to local language, cultural norms, seasonal disease trends or parents' immediate concerns at the clinic. This kind of responsiveness can improve understanding and build trust. However, our results indicate that variability becomes a problem when it leads to the omission of key vaccine messages, such as information on schedules, side effects, their management or follow‐up visits. We therefore advocate for a minimal core set of vaccine communication messages to be delivered consistently, while allowing flexibility in how these messages are framed, emphasised or discussed based on local context.

Traditional vaccine education has often focused on addressing a ‘knowledge deficit’, assuming that providing accurate information would increase vaccine uptake. However, research shows that vaccine hesitancy is influenced by emotional, cultural, social and political factors beyond cognitive understanding [33]. Vaccine communication typically follows either a presumptive approach, assuming readiness to vaccinate or a participatory approach, encouraging discussion [34]. In our study, most health talks (14/16, 87.5%) were non‐interactive, highlighting a gap in participatory communication. The WHO emphasises that effective communication involves asking open‐ended questions, reflecting concerns, praising positive health behaviours, advising clearly and checking for understanding. These elements align with newer, more structured participatory communication—open dialogue—techniques such as motivational interviewing [35]. However, open dialogue techniques are time and resource‐intensive and typically used in one‐on‐one settings. Efforts to simplify and combine motivational interviewing with presumptive communicative strategies are being explored [34], and further research is needed to apply them to group sessions.

Together, these findings highlight several pragmatic opportunities to improve vaccine communication in group clinic settings. At the national level, the National Primary Health Care Development Agency could develop a brief, standardised minimum curriculum for immunisation health talks that prioritises essential but inconsistently delivered messages, including vaccine schedules, common side effects and their management, vaccination during mild illness and the importance of vaccination cards and follow‐up visits. Health worker capacity could be strengthened through feasible, low‐cost approaches such as peer mentorship, supervisory refreshers and brief communication modules embedded within existing immunisation and maternal–child health programs. In addition, standardised recorded audio messages supported by pictorial guides or short pre‐recorded audiovisual health talks may offer a scalable option to ensure message consistency while reducing health worker burden in high‐volume clinics.

Although this study was conducted in two Nigerian states, many findings are likely applicable to other sub‐Saharan African settings where routine immunisation services depend on group clinic–based health talks within similarly constrained health systems marked by high client volumes and limited health worker time. At the same time, factors such as language diversity, sociocultural norms and health system organisation may affect how vaccine communication is delivered and received, underscoring the importance of maintaining fidelity to core messages while allowing local adaptations.

The study had several limitations. We used the secret shopper technique for one recording to minimise our influence on health workers' health talks, but our presence in the clinic likely still affected the interactions. We could not validate the nurses' training, so we cannot assess their training quality; however, most health workers noted that no formal training is provided. While parent interviews were private, social desirability bias may have influenced their feedback on the health talk. While content analysis provided a structured approach to assess the health talks, the absence of a standardised framework for vaccine communication necessitated the creation of a custom coding scheme, which may limit the analysis's replicability in other contexts. We did not obtain demographic data from parents who declined to participate, which may introduce selection bias and limit transferability. While qualitative studies do not aim for statistical generalisability, differences between participants and non‐participants should be considered when applying these findings to other settings.

## 5 Conclusion

In Nigeria, there is significant variability in the content of vaccine‐related health education during group clinic sessions. The topics discussed often depended on factors like scheduling or the facilitator's mood, resulting in missed information. Communication methods lacked structured training and tended to be either presumptive or participatory. There is an urgent need for evidence‐based strategies to improve vaccine communication and address vaccine hesitancy. Future implementation research should evaluate the effectiveness, feasibility and acceptability of standardised group‐based approaches, including recorded audio or audiovisual messaging with pictorial support, in settings where group clinic‐based health talks prevail.

## Funding

This project was funded with support from the Indiana Clinical and Translational Sciences Institute, which is funded in part by Award Number K12TR004415 from the National Institutes of Health, National Center for Advancing Translational Sciences, Clinical and Translational Sciences Award.

## Ethics Statement

The Borno, Cross Rivers State and Indiana University institutional review board ethical review board approved the study (Protocol # 97/2023, # CRSMOH/HRP/REC/2023/416 and # 20006).

## Consent

Each study participant gave written informed consent before any study activity.

## Conflicts of Interest

The authors declare no conflicts of interest.

## Supporting information


**Table S1:** tmi70101‐sup‐0001‐Supinfo.docx.

## Data Availability

The data that support the findings of this study are available on request from the corresponding author. The data are not publicly available due to privacy or ethical restrictions.
