# Supplementary material for: Content and Factors Influencing Health Education in Immunisation Clinics in Nigeria
Source: Trop Med Int Health. 2026 Feb 8;31(5):603–11. doi: 10.1111/tmi.70101 (PMC13139512; doi:10.1111/tmi.70101)
Supplement: Supplementary file 1 — Table S1: tmi70101‐sup‐0001‐Supinfo.docx. [file TMI-31-603-s001.docx]

**Supplemental Table:** Checklist for immunization-specific topics

| **Category** | **Item** |  |
| --- | --- | --- |
| **Vaccines** | | |
|  | Bacillus Calmette-Guérin (BCG) |  |
|  | Oral polio vaccine (OPV 0) |  |
|  | OPV 1 |  |
|  | OPV 2 |  |
|  | OPV 3 |  |
|  | Hepatitis B vaccines (Hep B) |  |
|  | Pentavalent vaccines 1 (Penta 1) |  |
|  | Penta 2 |  |
|  | Penta 3 |  |
|  | Rota virus vaccine 1 (Rota 1) |  |
|  | Rota 2 |  |
|  | Rota 3 |  |
|  | Inactivated polio vaccine (IPV 1) |  |
|  | IPV 2 |  |
|  | Pneumococcal vaccine (PVC 1) |  |
|  | PCV 2 |  |
|  | PCV 3 |  |
|  | Measles 1 |  |
|  | Measles 2 |  |
|  | Yellow fever |  |
|  | Meningococcal A vaccine (MenA). |  |
| **Vaccine administration age** | | |
|  | Birth to two weeks |  |
|  | Six weeks |  |
|  | Ten weeks |  |
|  | Fourteen weeks |  |
|  | Six months |  |
|  | Nine months |  |
|  | Twelve months |  |
| **Diseases vaccine present** | | |
|  | Tuberculosis |  |
|  | Polio |  |
|  | Hepatitis B |  |
|  | Diphtheria |  |
|  | Haemophilus influenzae type, b |  |
|  | Pneumonia |  |
|  | Rota Virus gastroenteritis |  |
|  | Measles |  |
|  | Yellow fever |  |
|  | Meningococcal meningitis |  |
|  | Whopping cough |  |
|  | Tetanus |  |
| **Common side effects** | | |
|  | Fever |  |
|  | Swelling |  |
|  | Scar formation |  |
|  | Site pain |  |
|  | Crying and fussiness |  |
| **Vaccine administration site** | | |
|  | Orally (mouth) |  |
|  | Left upper arm |  |
|  | Right thigh |  |
|  | Left thigh |  |
|  | Right upper arm |  |
| **Vaccination with mild illness** | | |
|  | Yes or No |  |
| **Importance of vaccine care** | | |
|  | Yes or No |  |
| **Miscellaneous** | | |
|  | Immunity booster (Vitamin A) |  |
